# Managing an altered social context—Patients experiences of staying away from home while undergoing proton beam therapy 

**DOI:** 10.1002/nop2.490

**Published:** 2020-04-14

**Authors:** Marie‐Louise Möllerberg, Ulrica Langegård, Emma Ohlsson‐Nevo, Per Fransson, Birgitta Johansson, Karin Ahlberg, Katarina Sjövall

**Affiliations:** ^1^ RI.SE (Research Institute of Sweden) Lund Sweden; ^2^ Institute of Health and Care Sciences Sahlgrenska Academy University of Gothenburg Gothenburg Sweden; ^3^ Faculty of Medicine and Health University Healthcare Research Centre Örebro University Örebro Sweden; ^4^ Department of Nursing Umeå University Umeå Sweden; ^5^ Department of Cancercentrum Norrlands University Hospital Umeå Sweden; ^6^ Experimental Oncology Department of Immunology, Genetics and Pathology Uppsala University Uppsala University Hospital Uppsala Sweden; ^7^ Department of Oncology Skåne University Hospital Lund Sweden; ^8^ Department of Oncology Lund University Lund Sweden

**Keywords:** brain tumour, interviews, nursing, proton beam therapy, psychological adaptation, qualitative method, radiotherapy, social context, social support, staying away from home

## Abstract

**Aim:**

To illuminate the experience of an altered social context for patients with primary brain tumours living away from home while undergoing proton beam therapy.

**Design:**

A descriptive, qualitative cross‐sectional interview study.

**Methods:**

Nineteen patients were interviewed between December 2015–August 2016, either during (*N* = 7) or before and after (*N* = 12) their proton beam therapy. A hermeneutical analysis was performed.

**Results:**

Participants made adjustments to achieve control and well‐being during the treatment period. The analysis also revealed two interrelated patterns that helped participants adjust: being part of the family from a distance and seeking affinity.

**Conclusion:**

It is important that patients receiving treatment far from home find a way to remain a part of their family and find affinity in the altered social context. Health professionals can prepare patients for the treatment period and can implement interventions to promote well‐being for both patients and their relatives.

## 1 INTRODUCTION

Living away from home for several weeks for medical treatment has become a reality for many patients in Sweden with primary brain tumours since proton beam therapy (PBT) has become an option for treatment. Proton beam therapy is an irradiation technique for patients with primary brain tumour, with the potential to reduce the non‐desirable radiation dose to healthy brain while having biological effects on the targeted tissue, comparable to conventional radiotherapy with photons (Thurin et al., 2018). The diagnosis of primary brain tumour is often associated with debilitating symptoms that limit patients' activities and opportunities in daily life, living away from home during treatment might pose an additional challenge for patients and their relatives.

### 1.1 Background

Every year, approximately 1,400 adults in Sweden are diagnosed with primary brain tumours. The disease can occur at any age, with a median age of 61 years in Sweden (The National Board of Health & Welfare, 2017). Radiotherapy is used to treat brain tumours, either as the primary treatment modality or as a supplement to surgery and/or chemotherapy (Wen & Kesari, 2008). In Sweden, there is one clinic that offers PBT; therefore, many patients need to travel a long distance to receive their treatment and stay in a hotel far away from home during the treatment period (5–6 weeks). A family member may accompany the patient at their own expenses.

Social support is an important factor in managing life circumstances for patients with primary brain tumour (Strang & Strang, 2001). However, given the complex cognitive and behavioural effects, people with primary brain tumour may find it difficult to maintain existing social groups and establish new ones (Klein et al., 2002). Social context is defined as the sociocultural forces that shape people's day‐to‐day experiences and directly and indirectly affect health and behaviour (Pasick & Burke, 2008). Adjusting to a new social context may take time because the relationship between individuals and their social context is dynamic, shaped and organized by social, cultural, economic, political, legal, historical and structural forces. Individuals are not always aware of the influence of social context on their lives (Burke, Joseph, Pasick, & Barker, 2009). Coming into a new social context alone to receive a life‐saving treatment is not always beneficial to patients' well‐being and quality of life. Previous studies reported that staying away from home for treatment is like “a cage of safety and discomfort” for patients with breast cancer receiving radiotherapy (Lilliehorn & Salander, 2018). Some used the time to socialize with their “fellow sisters,” while others isolated themselves and mainly found it burdensome to be there. In contrast, an Icelandic study found that staying away from home for treatment does not affect patients' psychological well‐being or the way they cope (Hjörleifsdottir, Rahm Hallberg, Ågren Bolmsjö, & Gunnarsdottir, 2007). None of these studies included patients with primary brain tumours.

Primary brain tumour is a complex and distressing disease that threatens a person's life and often changes the individual's personality (Strang & Strang, 2001), influencing the life situation for both patients and their family members. Most people with primary brain tumour experience functional or/and neurocognitive symptoms, poor mental function and decreased quality of life (Ownsworth, 2016). The disease is also associated with high level of psychological distress for both patients and their family members. Neurocognitive symptoms may influence the patients' ability to interact socially, especially with new persons in a new environment (Whiting et al., 2012). Many individuals with brain tumour are stricken with functional and psychosocial consequences resulting from the diagnosis or treatment (Langegard, Ahlberg, et al., 2019; Langegard, Johansson, et al., 2019; Ownsworth, 2016; Sherman et al., 2016; Simpson et al., 2015). Patients with primary brain tumour may also be in a more complex situation when travelling for treatment as a result of cognitive and behaviour symptoms. They experience many challenging symptoms that they need to manage during their treatment period, while alone and away from their family and ordinary social context. More knowledge is needed about patients' experiences of living away while undergoing PBT in a new social context.

## 2 THE STUDY

### 2.1 Aims

The aim of this study was to explore the experience of an altered social context for patients with primary brain tumours staying away from home while undergoing PBT.

### 2.2 Design

This study had a qualitative and explorative design. A secondary analysis was conducted using data collected from a previous interview study aiming to explore the process of symptom management in patients with brain tumour who received PBT (Langegard, Ahlberg, et al., 2019).

### 2.3 Method

#### 2.3.1 Setting

Patients who are referred for PBT travel to the Skandion Clinic in Uppsala from all over Sweden. There is an option for the patient to receive treatment at their home clinic with conventional radiation, even if they are recommended PBT. Many patients are unable to commute between the clinic and their homes and therefore stay in a nearby hotel during the treatment period. The treatment is given on a daily basis Monday through Friday over about 6 weeks, so the hotel stay may last 43–46 days. It is possible to bring a relative if needed. All meals are served in the residence restaurant, and there are lounges on each floor for common activities. There are no healthcare personnel. Financial compensation for patients differs, depending on the regulations in their county of residence. However, most receive compensation for at least accommodation and breakfast.

#### 2.3.2 Participants

Participants were referred for PBT from one of Sweden's seven university hospitals. The inclusion criteria were as follows: Swedish speaking, age 18 years or older, primary brain tumour diagnosis, receiving PBT and staying in a nearby hotel during the treatment period. Of the 22 participants from the original study population (Langegard, Ahlberg, et al., 2019), three participants were excluded because they lived at home during the treatment period; therefore, 19 patients participated in the current study (Table 1).

**TABLE 1:** Participant characteristics (*N* = 19)

	*N* (%)
Sex	
Male	11 (57.9)
Female	8 (42.1)
Age	
26–45	8 (42.2)
46–65	9 (47.3)
66–75	2 (10.5)
Education	
Elementary	3 (15.8)
Secondary	8 (42.1)
University	8 (42.1)
Civil status	
Married living with children at home	10 (52.6)
Married	4 (21.1)
Single	5 (26.3)
Distance from home, km	
100–400	9 (47.4)
401–600	7 (36.8)
601–750	3 (15.8)

#### 2.3.3 Data collection

The participants were strategically selected to provide a broad perspective, with selection based on age, sex and civil status. The participants were informed of the study and invited to participate by the second author (U.L) via telephone. Of the 19 participants, seven were interviewed during the treatment period and the remaining 12 were interviewed both before and after the treatment period, for a total of 31 interviews. The second author (U.L) conducted 25 of the interviews, and the remaining six interviews were conducted by an experienced oncology nurse. The interviews, which lasted from 30–70 min, were conducted at the Skandion Clinic or at two of the university hospitals between December 2015–August 2016. The interviews were recorded and transcribed verbatim by the second author (U.L). Five of the interviews were conducted by telephone, in accordance with the participants' wishes. The interviews started with an open question: “Can you please tell me about your situation based on your current illness, including how you manage the symptoms you experience?” Questions such as, “Can you tell me what it means for you to be away from home for 6 weeks?” and “How is it for you when you are away from your family during this 6 weeks?” were asked to invite participants to reflect deeper on their situation when away from home. Clarifying questions were also asked, for example, “Can you tell me more about that?”

### 2.4 Analysis

The interview data were analysed using hermeneutical analysis (Austgard, 2012; Fleming, Gaidys, & Robb, 2003) to interpret and understand the experiences of an altered social context for patients with brain tumour staying away from home while undergoing PBT. According to Gadamer (2004), new understanding emerges from openness, participation and dialogue. Through a dialectical process between the revealed experience and the researchers pre‐understanding, the text can be explored, examined and re‐examined. The process facilitates a “fusion of horizons” between the researchers and the text, which is essential for developing new understanding. During a hermeneutic inquiry, there is constant movement between the parts and the whole within the text as well as between the researchers and their pre‐conceptions throughout the interpretation process, with the aim of gaining new meaning and understanding (Austgard, 2012; Fleming et al., 2003; Gadamer, 2004). The last step in the hermeneutic process is the creation of new knowledge based on this revised understanding (Austgard, 2012). The first and second authors had the main responsibility of the analyse process with help from the research team (consist of one PhD student, one junior researcher and five senior researchers with experience of qualitative methods). Initially, the interviews were repeatedly read with openness to get a sense of the whole and to search for the essential meaning of the text. Then, each interview was reread to identify and gain an understanding of recurrent experiences. The research team read the text separately and together, reviewed, discussed and refined the analysis. Similar experiences were clustered into themes. One overall theme emerged through this process, “adjustments to an altered social context.” The research team re‐examined the interviews with the identified theme in mind, focusing on participants' experiences and questioning their own prior understandings. To gain an increased understanding, the research team moved back and forth between looking at sections of the text and relating them to the meaning of the whole text. The analysis was reviewed, discussed and refined by the research team to ensure credibility. Throughout the analysis, every attempt was made to keep questioning open and to challenge interpretations.

#### 2.4.1 Rigour

To ensure the trustworthiness of the study, we used a self‐conscious and reflective approach throughout the data collection and analysis to ensure dependability (Polit & Beck, 2012). Knowledge gained from participant interviews was combined via a constant cyclic process to attain an in‐depth exploration and understanding of the study aims. Credibility was ensured through the audio recording of all interviews followed by accurate transcribing. The interviewer strived to be non‐judgemental and neutral to all responses from the participants, and the questions were asked in as neutral a manner as possible. Areas of disagreement required re‐examination of the original data, and further discussion until agreement was reached. Any pre‐conceptions of the studied phenomenon were continuously addressed and made explicit, with the aim of being open to explore the participants' experiences.

### 2.5 Ethics

In accordance with the Declaration of Helsinki, all participants received oral and written study information and provided written informed consent to participate (World Medical Association, 2013). Talking about the experience of having cancer might cause anxiety or bring about other psychosocial problems for the person with cancer. If such need arose during any interview, there was possibility to facilitate psychosocial support. The research committees at all involved hospitals and the Regional Ethical Review Board in Gothenburg, Sweden, no. 433‐15, approved this study.

## 3 RESULTS

The findings are presented with quotations from the participants, and text between brackets is authors' clarifications.

### 3.1 Managing an altered social context

Hermeneutic analysis revealed a process within participants where they made several adjustments to achieve control and well‐being during the treatment period. This process enables participants to better cope with their own worries and demands, their family members' worries and demands at home and their new social context. The process started with the decision to undergo PBT and for some participants ended when they returned home and differed from participant to participant. Some remained worried throughout the entire treatment period for one or several different reasons, while others were mostly worried before the PBT started. They were worried about the treatment and the symptoms that may occur, how they would manage moving into an altered social context in a new environment (hotel), with new people (including hotel staff, health care personnel and other patients), in a new town (sometimes visited for the first time). Participants were often alone and away from their families and thus had little or no access to their usual social and practical support, which may have increased their worry. They also had thoughts about how sick the other patients were and if there would be someone with whom they could spend time. The participants' concerns about having someone to spend time with had various causes (e.g. do not want to be alone, exchange of experiences, social support to help manage their treatment or to help pass the time). Therefore, they were forming new acquaintances with other patients who had similar experiences. After treatment started, participants worried more about how their families were managing at home and experienced shame and guilt because they were not able to help with the practical things at home. At the same time, they were aware that their family members worried about them and how they were coping, which may have increased participants' feelings of guilt. In addition, all participants had a life‐threatening diagnosis, which increased their worry and thoughts about the future. The participants felt a bit relieved when they could start looking forward to and preparing for life at home again. Feelings surrounding safeness, reconnaissance, knowing the rules and having their loved ones close which helped participants to achieved well‐being. Before the treatment started, 6 weeks felt like a long time, but after treatment was finished, 6 weeks felt like a short time.

The analysis also revealed two patterns that helped participants adjust: *being part of the family from a distance* and *seeking affinity.*

### 3.2 Being part of the family from a distance

Participants felt insecure while away from the family during the treatment period because the separation prevented them from receiving the support, and they were used to having from their family members. It was hard to be away from home because they needed to see that their family was doing well and managing daily tasks. Daily contact with their family strengthened the feeling of being included and helped them to maintain normality. Separation from their children was the most burdensome and contributed to feelings of guilt—they felt they had abandoned their children but at the same time they were aware that the treatment would allow them to live together with their family in the future. Participants also felt frustrated that they could not help their partners with daily tasks, and they experienced that their partners at home expressed frustration and worries, which increased the participants' sense that they had abandoned their families and added to their sense of guilt. Experiencing these feelings during the treatment period decreased their well‐being:

So, I get a bad conscience because of the children when I am not at home together with them. And even for my wife that I cannot be at home and help her. (1121)

The family structure influenced how the participants dealt with the situation. If the participants previously had control over the practical arrangements at home and planned for the family, this tended to increase their frustration regarding losing control of the daily tasks at home. Patients who lived alone at home did not seem to experience a such loss of control of the daily tasks at home, during treatment. Participants with families created new routines to become part of the family, such as daily Skype conversations, with some participants doing homework together with their children via Skype. The hardest part was preparing for the treatment period because participants did not know how they were going to feel or what symptoms might appear. Both the participants and their families needed a plan to help them manage during treatment and afterwards. It was important to develop the plan together and to plan as much as possible before the treatment started. All participants looked forward to getting treatment for the brain tumour started as soon as possible:

She (talking about his wife) is a very strong person; she also feels relief that it (treatment) finally should be started. We had a plan in advance how it is going to be. (1016)

The forced separation from family and being alone were the most frightening things associated with treatment away from home. However, staying at the hotel during treatment was experienced with contradictory feelings. Some participants used this time away from home to focus on themselves and figure out their own needs, possibly increasing their well‐being. Having time for oneself was also related to feeling of guilt. Their expectations of themselves and their loved ones differed from when they lived at home, where they may have prioritized needs of family members above their own needs. Not having immediate responsibility for anyone else could be a relief:

When I am at home, I have more expectations on myself to be part of the family and to be working. At home it is not allowed to just sleep or rest. But here I can do that. (1095)

### 3.3 Seeking affinity within an altered social context

It was important for participants to start new relationships in an altered social context. Even if they kept in touch with family and friends via telephone or Skype, they needed to have someone to talk with—just to feel that they were not alone was calming. A common need was to feel affinity with other patients and to have someone to talk to who understood what you were going through. It was a relief to meet other patients in a similar situation, just to confirm that the feelings and thoughts they were experiencing were normal:

It is important to have social contact with others, to not be alone and it is not possible to be alone for 5 weeks, no it is not possible, you have to have someone to talk to. All of us are the same, everybody is seeking contact. (1087)

Meeting patients that had already experienced a week or two of treatment and were still managing alright alone increased participants' sense that all was going to be fine. The patients helped each other get included into groups at the hotel, and hotel staff also helped participants introduce themselves to other patients. Some participants chose to be alone and did not seek affinity with other patients, and some participants found it easier than others to connect with new people. They also found that their fellow patients met different needs than their family did, because they had their own experiences of illness and treatment and could share it, providing advice to other patients, including how to manage and live with symptoms during the treatment period:

It is like therapy to be there (at Skandion). To have the opportunity to talk and discuss with people who really understand what it means (to receive PBT). (1104)

## 4 DISCUSSION

Living at a hotel, often alone, during PBT was an additional challenge for patients with primary brain tumour, who were already handling a complex situation with many challenges, such as cognitive and behavioural symptoms. The participants experienced an ongoing process were they made several adjustments to cope, achieve well‐being and manage the altered social context. This study revealed an overall theme: *managing to an altered social context* and two interrelated patterns: *being a part of the family from a distance* and *seeking affinity within an altered social context.*

Our findings revealed that families played an important role in providing social support and patients made adjustments to be a part of the family from a distance on a daily basis. This finding is consistent with earlier studies (Harrop et al., 2017; Payne, Jarrett, Jeffs, & Brown, 2001; Strang & Strang, 2001). Previous studies also found that close relationships with children, family or friends give meaning to life and give patents the strength to struggle through (Kvåle & Synnes, 2013; Strang & Strang, 2001). Our results showed that patients made adjustments to keep daily contact and be involved with their families (e.g. helping with homework via Skype) and maintained the ability to receive social support from and give social support to their families. This adjustments may explain the findings of earlier studies that patients living away from home during treatment experienced greater support and feelings of intimacy with their loved ones (Payne et al., 2001) and that living away does not always seems to negatively affect patients' psychological well‐being (Hjörleifsdottir et al., 2007). However, participants' separation from their families gave an opportunity to focus on their own needs which sometimes raised feelings of guilt about abandoning their children or family, which has also been found in other studies where participants reported that staying away from home was a waste of time (Lilliehorn & Salander, 2018) or that they were frustrated because they knew they were needed at home (Hegney, Pearce, Rogers‐Clark, Martin‐McDonald, & Buikstra, 2005). This study and a previous study (Langegard, Johansson, et al., 2019) showed that some patients were worry before and especially at the beginning of the treatment period. This is in line with previous studies that showed that being away from home and separated from loved ones meant additional worry for both patients and their families (Payne et al., 2001). In contrast, Fitch et al. (2003) found that being away from home could be valuable because it reduced the burden on family members. Our findings that seeing the time away from home as a relief, when they could focus on themselves, are in line with an earlier study where participants described it as a blessing because it provided a break from a burdensome life at home (Lilliehorn & Salander, 2018). Staying away from home could be experienced in both positive and negative ways; therefore, it is essential to identify the individual needs of support of each patient.

Our study revealed an ongoing process where participants made adjustments to achieve control and well‐being during the treatment period to cope with the altered social context. This could be understood in the context of Albert Bandura's social cognitive theory, where a person's behaviour and the environment influence each other (Bandura, 1986). When people interact with each other, the context where their actions take place affects their behaviour. Our understanding of words, emotions and social cues may differ depending on where we encounter them. Our study showed that the altered social context influenced participants to make adjustments to feel a bit of normality and find affinity. This illuminates the significance that social context has on patients' behaviour, which is in line with Burke et al.'s (2009) finding that research needs to include social context to gain a deeper understanding of behaviour to achieve health. The participants in the present study experienced another kind of support when they talked to other patients in a similar situation. Sterckx et al. (2013) reported that talking with other patients about symptoms creates expectations and helps individuals cope with their situation. This raises the question whether support groups where patients could talk to other patients and/or family health conversations could promote family well‐being and increase patients' sense of affinity. Further research is needed to evaluate this kind of intervention.

### 4.1 Limitations

The present study has some limitations regarding the sample, data collection and analysis. Patients who were having difficulty adapting to the treatment and to staying away from home may have refused to participate. Furthermore, the patients were predominantly of Swedish origin and various cultural perspectives were not addressed; therefore, transferability to other contexts cannot be determined. Most of the sample was younger than 65 years, highly educated, married and living with children at home, which may not be representative participants.

This study used interview transcripts gathered for another study (Langegard, Johansson, et al., 2019). The secondary analysis of the present study was performed as some data were not presented earlier; and as it turned out, the experiences of being away from home were expressed by the participants to be of importance in various degrees. However, data collection might be some limited because of this. To handle a possible limited data collection, the researcher who conducted most interviews played an active role in the present study. Although it was not the primary focus, several follow‐up questions about being away from home were asked during the interviews.

## 5 CONCLUSION

When patients face being away from home during several weeks of treatment, it is important they find a way to still be a part of the family and to achieve a feeling of affinity in the altered social context. Knowledge regarding the influences of an altered social context for patients with brain tumours who are away from home while undergoing PBT is important for health professionals working at both the distant clinic and the patient's home clinic. Knowledge gained from this study can help health professionals at the home clinic to better prepare patients with necessary information for treatment at a distant clinic and help health professionals at the distant clinic to develop interventions to promote well‐being for both patients and their relatives.

## CONFLICT OF INTEREST

No conflict of interest has been declared by the authors.

## AUTHOR CONTRIBUTIONS

M‐LM, UL, EON, PF, BJ, KA and KS made substantial contributions to conception and design, or acquisition of data, or analysis and interpretation of data, involved in drafting the manuscript or revising it critically for important intellectual content, given final approval of the version to be published and agreed to be accountable for all aspects of the work in ensuring that questions related to the accuracy or integrity of any part of the work are appropriately investigated and resolved. Each author should have participated sufficiently in the work to take public responsibility for appropriate portions of the content.
